# Supplementary material for: Patterns of Intron Gain and Loss in Fungi
Source: PLoS Biol. 2004 Nov 30;2(12):e422. doi: 10.1371/journal.pbio.0020422 (PMC532390; doi:10.1371/journal.pbio.0020422)
Supplement: Table S1 — Also available at http://genes.mit.edu/NielsenEtAl/. (4.3 MB ZIP). [file pbio.0020422.st001.zip › NielsenEtAl/html/394.html]

AN4178.1.NCU03859.1.MG07266.1.FG09989.1


```
 CLUSTAL W (1.82) Multiple Sequence Alignments - Introns Inserted


Sequence 1: MG07266.1	366 aa
Sequence 2: FG09989.1	349 aa
Sequence 3: NCU03859.1	355 aa
Sequence 4: AN4178.1	359 aa
Alignment Length: 381 aa
Number Identitical Residues: 159 aa
Alignment Score (without introns) 8214


MG07266.1 	-MAESAAKRLKTEGGSSEGPVTIGTHN~GHFHADEALAVYMLRQHIPTYAGASLVRTRDP
NCU03859.1	-MAENAAKRIKTDG----GALTIGTHN~GHFHADEALAVHMLRQHVPKYTGARLVRTRDP
FG09989.1 	-MAESAAKRVKTSG---NGPL-IGTHS~GHFHADEALAVHMLRR-LPTYRDADLVRTRDP
AN4178.1  	MATESVSKKPRIDS------PTIGTHN2GHFHADEALAVYLLRL-LPAYSQSPLIRTRDP
          	  :*..:*: : ..       :****. ***********::**  :* *  : *:*****

MG07266.1 	AALARCHTVVDVGGEYDASTLRFDHHQRTFSTTFPGRQTKLSSAGLVYMHFGKQIIARRL
NCU03859.1	ALLETCDIVVDVGGEYEPARDRYDHHQRTFTTTFPDHQTKLSSAGLVYMHFGKGIISRSL
FG09989.1 	AVLATCHTVVDVGGEYDAEKRRFDHHQRGFNTTFPGRPTKLSSAGLVFLHFGRAIVAERL
AN4178.1  	ALLQTCHTVVDVGGEYDAATNRYDHHQRTFNSTFPGYKTKLSSAGLVYLHFGQSIIANHA
          	* *  *. ********:.   *:***** *.:***.  *********::***: *::.  

MG07266.1 	ADPDCDNEATVDMLYAKLYENFVEALDAHDNGISAYDPAVLATASNGQPLQKRFSDGGFT
NCU03859.1	PDAPAEDNDKVGLIWNKLYDSFVEALDAHDNGISVYDSDALKAAG----IEKRFSNGGFT
FG09989.1 	G--LSEDSPDVDLIYKKLYENFVEALDAHDNGISVYDPAAIAAAG----IEKRFSEGAFG
AN4178.1  	S--LPADHSNVSLIFNKLYADFIEAIDANDNGIGVYDPEKLSEAG----IEKRFKDGGVT
          	      :   *.::: *** .*:**:**:****..**.  :  *.    ::***.:*.. 

MG07266.1 	LGAVVGRLNPNWNDPIPEDPAEAQAAEDQRFEAASARIGEEFDRDLDYYTRSWLPARSVV
NCU03859.1	LGAMVGRFNGNWNDTAPSDPVAAQAEEDGRFEQASQRIGEEFDRALDYYTKAWLPARDVV
FG09989.1 	LGAVVGRLNPKWNDPTPSDPAEAQAAEDAKFNEASSRIGQEFDRDLDNYAASWLPARTIV
AN4178.1  	IASIVNDMN----TPSPED-EAAGIDEDGLFNQASQFVGSVFSRKLRHAVNSWLPARETV
          	:.::*. :*     . *.*   *   **  *: **  :*. *.* *   . :*****  *

MG07266.1 	ADAFSHRAEHDPAGKGRILVFKGQSVPWKDHLYTLEQE--------NPSAGEVVYVLYPE
NCU03859.1	ETAYKARKEYD--AEGRILVLKGQSAPWKDHLYTLEEEE-------GERVQKVLYVLYPE
FG09989.1 	QEAFNKRKQYD--EQGRILILEGQSVPWKDHLYTLEDG--------TPS---VIYVLYAE
AN4178.1  	QAAYTDRKNVH--ASGKIMVLPQGGVPWKEHLYNFEKEAKAANINGNAEDAEVVYVLYPE
          	  *:. * : .   .*:*:::   ..***:***.:*.  .::. ..     .*:****.*

MG07266.1 	KPTPDAKWRIQCVPVSKDSFQSRLPLPEAWRGFRDEELDKITG------IPGCVFVHAAG
NCU03859.1	KPTPDAKWRVQCVPETGDSFQSRKPLPEAWRGFRDEKLDEITQ------IQGGVFVHAAG
FG09989.1 	KPEPGAKWRIQCVPESKDSFTSRKPLPEAWRGFRDAELDGISG------IPGCVFVHAAG
AN4178.1  	SAAEGSKWRVQAVSVNEGSFVSRKPLPEKWRGVRDADLDGVLAGEGAGIPEGAVFVHASG
          	..  .:***:*.*. . .** ** **** ***.** .** :  ...:.   * *****:*

MG07266.1 	FIGGNKTFEGAMAMAQKALELA
NCU03859.1	FIGGNKTFDGALAMAKKALE--
FG09989.1 	FIGGNKTFEGAKEMATKALEG-
AN4178.1  	FIGGHKTREGAFAMAAKALE--
          	****:** :**  ** ****
```
